# LectomeXplore, an update of UniLectin for the discovery of carbohydrate-binding proteins based on a new lectin classification

**DOI:** 10.1093/nar/gkaa1019

**Published:** 2020-11-11

**Authors:** François Bonnardel, Julien Mariethoz, Serge Pérez, Anne Imberty, Frédérique Lisacek

**Affiliations:** Univ. Grenoble Alpes, CNRS, CERMAV, 38000 Grenoble, France; Proteome Informatics Group, SIB Swiss Institute of Bioinformatics, CH-1227 Geneva, Switzerland; Computer Science Department, University of Geneva, CH-1227 Geneva, Switzerland; Proteome Informatics Group, SIB Swiss Institute of Bioinformatics, CH-1227 Geneva, Switzerland; Computer Science Department, University of Geneva, CH-1227 Geneva, Switzerland; Section of Biology, University of Geneva, CH-1205 Geneva, Switzerland; Univ. Grenoble Alpes, CNRS, CERMAV, 38000 Grenoble, France; Univ. Grenoble Alpes, CNRS, CERMAV, 38000 Grenoble, France; Proteome Informatics Group, SIB Swiss Institute of Bioinformatics, CH-1227 Geneva, Switzerland; Computer Science Department, University of Geneva, CH-1227 Geneva, Switzerland; Section of Biology, University of Geneva, CH-1205 Geneva, Switzerland

## Abstract

Lectins are non-covalent glycan-binding proteins mediating cellular interactions but their annotation in newly sequenced organisms is lacking. The limited size of functional domains and the low level of sequence similarity challenge usual bioinformatics tools. The identification of lectin domains in proteomes requires the manual curation of sequence alignments based on structural folds. A new lectin classification is proposed. It is built on three levels: (i) 35 lectin domain folds, (ii) 109 classes of lectins sharing at least 20% sequence similarity and (iii) 350 families of lectins sharing at least 70% sequence similarity. This information is compiled in the UniLectin platform that includes the previously described UniLectin3D database of curated lectin 3D structures. Since its first release, UniLectin3D has been updated with 485 additional 3D structures. The database is now complemented by two additional modules: PropLec containing predicted β-propeller lectins and LectomeXplore including predicted lectins from sequences of the NBCI-nr and UniProt for every curated lectin class. UniLectin is accessible at https://www.unilectin.eu/

## INTRODUCTION

Glycans, present as part of glycoconjugates at the surface of all living cells, are involved in cellular communication and a range of biological processes. Carbohydrates are therefore considered as ‘the third alphabet of life’, with information encoded in their composition, their complex structure and their dynamics (1). Lectins, are proteins able to decipher the so-called glycocode, they are defined by their functions since they contain one or more non-catalytic domains able to specifically bind mono or oligosaccharides (2). Glycan–lectin interactions play a crucial role in many biological processes, and are considered as hot-spots for therapeutic strategies (3). Lectins are also used for biotechnology and diagnostic: they are able to decipher fine differences in glyco-phenotypes of tissues, giving therefore crucial information for personalized medicine (4). Lectins occur in all branches of the living kingdoms, and the variety of their origins and folds challenges their classification. Previous attempts to classify lectins upon their origin or specificity do not reflect the subtle functional differences and versatility of these proteins (2). Among the recent suggestions, a grouping into 48 families based on fold and Pfam families (5) was detailed (6).

Glycobioinformatics databases and tools are increasingly being developed as described in recent reviews (7,8). Yet, only a limited number are devoted to support the study of lectins and their interactions with glycoconjugates. SugarBindDB describes adhesins and glyco-targeted toxins of microbial pathogens (9). The Lectin Frontier Database (LfDB) contains affinity data based on frontal affinity chromatography (10). The lectin pages in GlyCosmos (11) list protein entries annotated as lectins in UniProt. Information on lectins are also available from databases with a broader view on protein-carbohydrate interactions, such as ProCarbDB, a database of carbohydrate-binding proteins (12), the Database of Anti-Glycan Reagents (DAGR) that covers glycan-targeted antibodies and lectins as reagents (13), and the carbohydrate binding modules (CBM) pages in CAZy (14). The complete list of resources is listed in Supplementary Table S1.

The UniLectin portal was created in 2018, with the Unilectin3D module dedicated to lectin 3D structures and lectin/glycan complexes (15,16) and providing 1740 structures covering 428 different lectins and 765 references (as of 29 August 2018). UniLectin3D was confirmed as the main source of information on 3D structures of lectins and their interactions with ligands, as pointed in recent glycomics reviews (7,17). It has also been recognized as a resource complementing new tools for determining the specificity of proteins (12,18), as well as for analyzing glycophenotypes in personalized glycomedicine (4,19,20).

In order to extend UniLectin, the first focus was on lectins containing tandem repeats and resulted in launching the PropLec module, for the identification of β-propeller lectin candidates accurate on every blade composing the propeller. In PropLec, candidate lectins were identified from conserved motifs distinguishing six β-propeller lectin classes. The November 2019 UniProt release was predicted to contain 4877 β-propeller lectins. The prediction was validated by the identification and structural characterization of a novel type of propeller assembled by dimerisation of 3-blade repeat (21).

The latest module named LectomeXplore describes candidate lectins identified in available proteomes from all kingdoms and for all available lectin classes. These predictions are not provided in other lectin-related databases. Both PropLec and LectomeXplore are based on screening the UniProt (Swiss-Prot + TrEMBL) and NCBI-nr (non-redundant) protein databases to identify lectin candidates. The LectomeXplore module is based on a new lectin classification that was built from a collection of conserved motifs. This classification is composed of 109 lectin classes derived from 35 distinct folds detailed in (22). Each class is defined with a Hidden Markov Model (HMM) sequence profile generated from a manually curated protein alignment with HMMER-hmmbuild (23). Screening was performed with the HMMER-hmmsearch followed by post processing described further in this article and in (22). The new LectomeXplore module is introduced here in detail and illustrated with examples.

## UPDATE OF THE UNILECTIN PORTAL AND NEW CLASSIFICATION OF LECTIN STRUCTURES

The UniLectin3D module now includes 2225 3D-structures from 535 different lectins, corresponding to an increase of more than 25% in the last 2 years. Searching was improved by simplifying the graphical interface and adding a ‘glycan search’ to query the database by specificity, i.e., by monosaccharides and oligosaccharides bound to the lectin structures. The protein topology provided by the PDBe (24) is now available. External links to the full glycan structures containing the epitope recognised by the lectin as recorded in GlyConnect (25) as well as to matching lectins in SugarBindDB (9) are now available on each structure page. The PropLec module was added recently. It contains six classes of β-propellers, altogether resulting in the prediction of 4877 candidate lectins in protein sequence databases.

**Figure 1.**
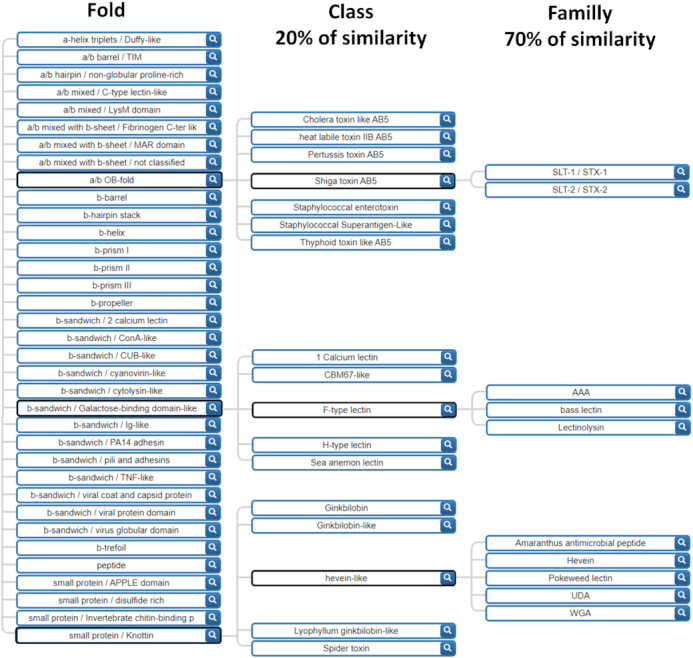
Hierarchical tree of the UniLectin3D lectin classification based on both sequence and structure. The tree is expanded for the Shiga toxin, F-type and hevein-like lectin classes in order to exemplify the variety of classes and families.

In order to characterise conserved motifs in lectin sequences, a new classification that accounts for different levels of sequence similarity, was needed. We ruled out using taxonomy as a classification criterion since low sequence similarity exists in some lectin families across kingdoms, such as the F-type lectins that are conserved from bacteria to mammals (26). In contrast, folds are more conserved than sequences and offer a more relevant criterion for building a classification. A great diversity of structures has risen by natural selection, and >30 distinct fold units have been observed for lectins. In each of the large families of folds based on α-helices, β-sheet and mixed structures, different patterns emerge by combinations. Out of 40 architectures (fold families) defined in CATH (27) only 17 are represented in UniLectin3D. In principle, any protein fold could evolve into a functional lectin under evolution pressure (28) however, most known lectins adopt an architecture consisting of β-sheets that assemble frequently in β-sandwiches, β-propellers, β-barrels and β-prisms. Higher symmetry folds are also frequent in lectins and bring multivalence properties of multiple glycan-binding on cell surfaces. The new classification is organized on three levels: (i) **the fold level** characterizes the whole lectin domain (β-helix, β-propeller, etc.) as derived from the protein 3D structure. The nomenclature is compliant with the reference structural databases CATH (27) and SCOPe (29) as well as with a structural lectin classification previously suggested (6); (ii) **the class level** is defined by sequence similarity having a 20% cut-off between different classes; (iii) **the family level** defined at a minimum of 70% of sequence identity. Manual inspection of structures of the UniLectin3D database (15) was necessary. For example, the lectin domain of proteins with multiple domains were manually selected to extract only the sequence of interest and eventual peptide tags were removed. More generally, a list of disqualifying domains, corresponding to non-lectin modules, was defined for further systematic removal. In the end, the classification resulted in 35-fold, 109 classes and 350 families as partially shown in Figure 1 featuring all folds and a selection of classes and families. All the lectin classes for each fold are listed in Supplementary Table S2.

UniLectin3D sequences are assigned to a class if they share 20% similarity with any sequence of that class. The class similarity cut-off was selected based on CATH classification parameters (27). The family cut-off was empirically selected to optimally group proteins with the same name into one family (i.e. Pili adhesins). Sequence similarity is computed with BlastP, launched with default parameters, a manually set *e*-value threshold of 10^−3^ and no coverage threshold. Results were checked manually. The division of the lectin of a same fold in multiple classes at 20% of similarity is illustrated with the fold β-sandwich/ConA-like in Supplementary Figure S1, which represents the disparity in term of number of structures by class and in the inner sequence conservation of each lectin classes.

## CONSERVED MOTIFS AND CONSTRUCTION OF THE LECTOMEXPLORE MODULE

Within each class, the lectin sequences were aligned with the Muscle software (30). Sequence redundancy was automatically discarded. The conserved regions resulting from the multiple alignments were then fed to a Hidden Markov tool with the HMMER-hmmbuild tool (31) to generate characteristic profiles of each lectin class, using default parameters except a symfrac set at 0.8. An HMM sequence profile is a probabilistic model which encodes amino acid conservation at each position in a defined region. A typical example is displayed in Supplementary Figure S2, representing the conserved motif of the F-type lectin class.

Using these 109 profiles, potential lectin sequences were searched in UniProtKB (UniProt April 2019) (32) and NCBI-nr (non-redundant July 2019) (33). Protein datasets were screened with all HMM profiles using HMMER-hmmsearch, with default parameters and a *P*-value below 10^−2^. The probabilities of substitution of each amino acid by any other are stored in the BLOSUM62 substitution score matrix that was used for the generation of the profiles **(**Supplementary Table S1**: databases and tools)**. Further filtering was applied to the cases of very similar sequences (multiple strains of the same species, natural mutation, sequencing errors, …). A post-processing step led to identify a single representative whenever proteins of the same species have identical 100 consecutive amino acids. Domains shorter than 10 amino acids were also filtered out. An HMMER bit score is generated for each predicted lectin. Because each family profile is generated independently of one another, the score values are not comparable across motifs used in the prediction. As a result, it is impossible to use a single cut-off value for all lectin classes. For the sake of simplicity, LectomeXplore provides a score easy to understand (between 0 and 1). The LectomeXplore normalized prediction/similarity score reflects the similarity between a prediction and the matched profiles. It is generated to rank the results and select the top one, as previously described in (22).

## OVERVIEW OF THE LECTOMEXPLORE MODULE

LectomeXplore supports multiple ways to explore the predicted lectins with entries based on the taxonomy, the lectin class, the protein name and other identified functional domains by Pfam. The LectomeXplore homepage provides an overview of the predicted lectins with a score over 0.5. Expanding the search with the use of lower score is possible via the advanced search page, which provides a broad set of criteria. A tutorial is available at https://unilectin.eu/predict/tutorial.

The distribution of the predicted lectins (with a score >0.5) spans 44186 proteins in Eukaryotes, 31 589 in Metazoa, 7780 in Viridiplantae, 4081 in Fungi, 8180 proteins in Viruses (including 4044 in Influenza) and 6943 proteins in Bacteria. In comparison, the NCBI-nr protein datasets contain 16 million proteins in Eukaryotes, 9 million in Metazoa, 8 million in Viridiplantae, 10 million in Fungi, 1 million proteins in Viruses and 116 million proteins in Bacteria. Some species, such as *Escherichia coli*, cover a large number of sequenced strains with similar proteins and after removal of the redundant protein sequences, 2076 predicted lectins remain in *E. coli* (based on 20922 strains of *E. coli* in the NCBI-nr dataset). Interestingly, even with a high score threshold most of the predicted lectins are annotated as Uncharacterized proteins (4403/59424 for a score of 0.5). Two classes of predicted lectins were very populated due to the high number of false positives, i.e. proteins wrongly identified as lectin candidates. TIM lectins, that are closely related to glycosylhydrolases, and Variable Lymphocyte Receptor (VLR), that are adaptative immune receptors from jawless fishes able to bind to a large number of antigens, were then excluded from whole proteome predictions. In other words, only 107 classes are used in all predictions.

LectomeXplore was assessed using the UniProt/Swiss-Prot curated protein dataset. Among 622 reviewed proteins with ‘lectin’ in their name, 86 were not predicted, which is expected since the motifs cover only lectins with known 3D-structures. In contrast, only 50 Galactose-related enzymes were predicted out of 719 enzyme entries where ‘galac’ is contained in the protein name. Overall, LectomeXplore has a sensitivity of 0.86 and a specificity of 0.93.

Shared cross-references to PDB between UniLectin3D and Pfam pointed at Pfam domains for which the associated annotation included the ‘lectin’ or ‘carbohydrate-binding’ terms. Those Pfam domains listed in Supplementary Table S3 are overlapping with lectin domains and were filtered out of LectomeXplore. The majority (92/105) of annotated Pfam families do mention these terms or related terms including ‘adhesion’, ‘glycan-binding’, ‘membrane-binding’, ‘coat-binding’ or ‘toxin’. Furthermore, Pfam contains DUFs (Domains of Unknown Function) that await proper annotation. We found at least two DUFs that match our lectin annotation. DUF3421 is likely to be an Oyster lectin and DUF346 a PLL-like lectin (PropLec7A).

**Figure 2.**
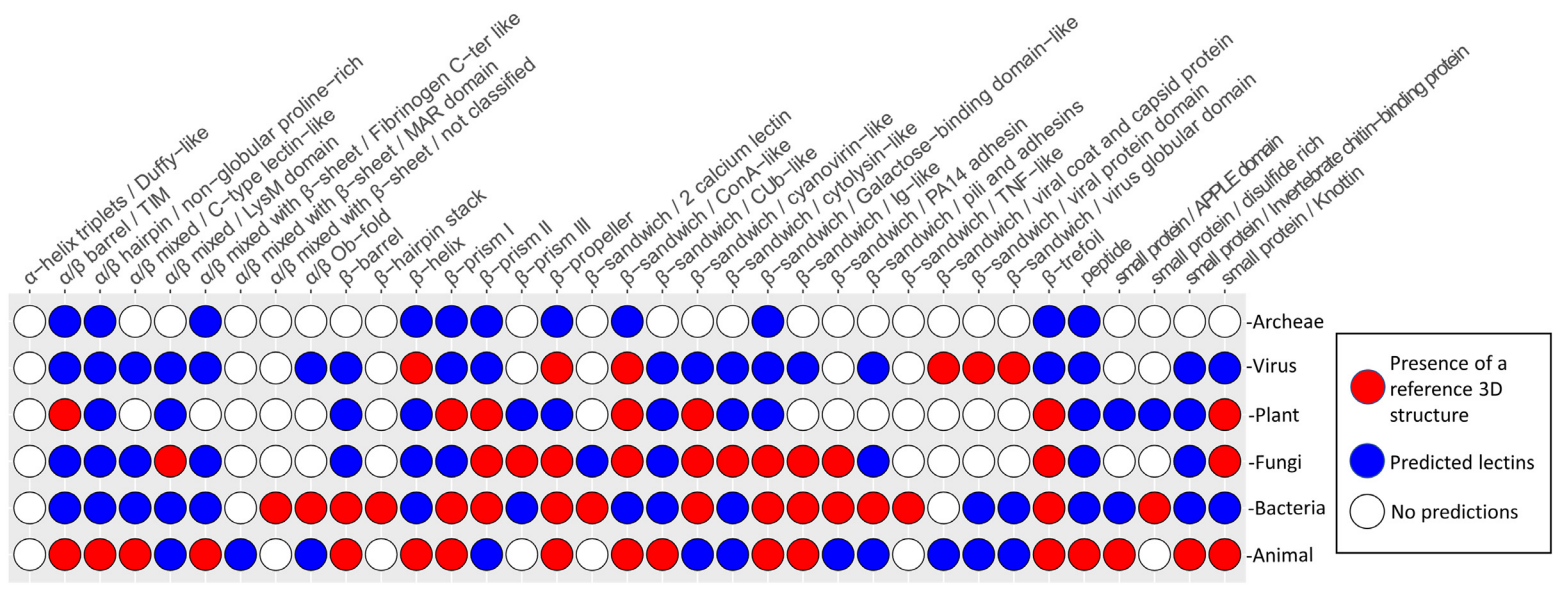
Distribution of curated versus predicted lectin folds across the kingdoms of life. Red circles represent the presence of reference 3D structures, blue circles represent the predicted lectins. In white are represented the fold not yet discovered for the corresponding kingdom.

For a large number of folds, 3D structures are available only in limited organisms, spanning only one part of the living kingdoms (Figure 2). However, the prediction here shows that most lectin folds have similar sequences all across the living species, acquired either by evolution from the same common ancestor, by gene transfer, or by natural selection towards a very similar structure. This information is available from the ‘Browse the predicted lectins by fold and origin’. For example, a β-prism III lectin only structurally characterised in fungi, is found in 40 actinobacteria of the *Streptomyces* genus.

## EXAMPLE OF LECTOME EXPLORATION

The lectome of any organism with a complete proteome can be investigated and compared to other lectomes. Considering species with top numbers of predicted lectins (Supplementary Table S4), many aquatic organisms, fishes and molluscs, are listed and appear to produce at least 20 lectins. Among them, cnidarians, such as sea anemones and corals, have large lectomes that have been shown to be involved in innate immunity (34). Indeed, these immobilised marine animals filter large amounts of sea water and use lectins for aggregating bacteria. Interestingly, large panels of different sugars can be recognized by the lectome of these invertebrates, probably linked to the ability to bind a variety of different bacteria.

**Figure 3.**
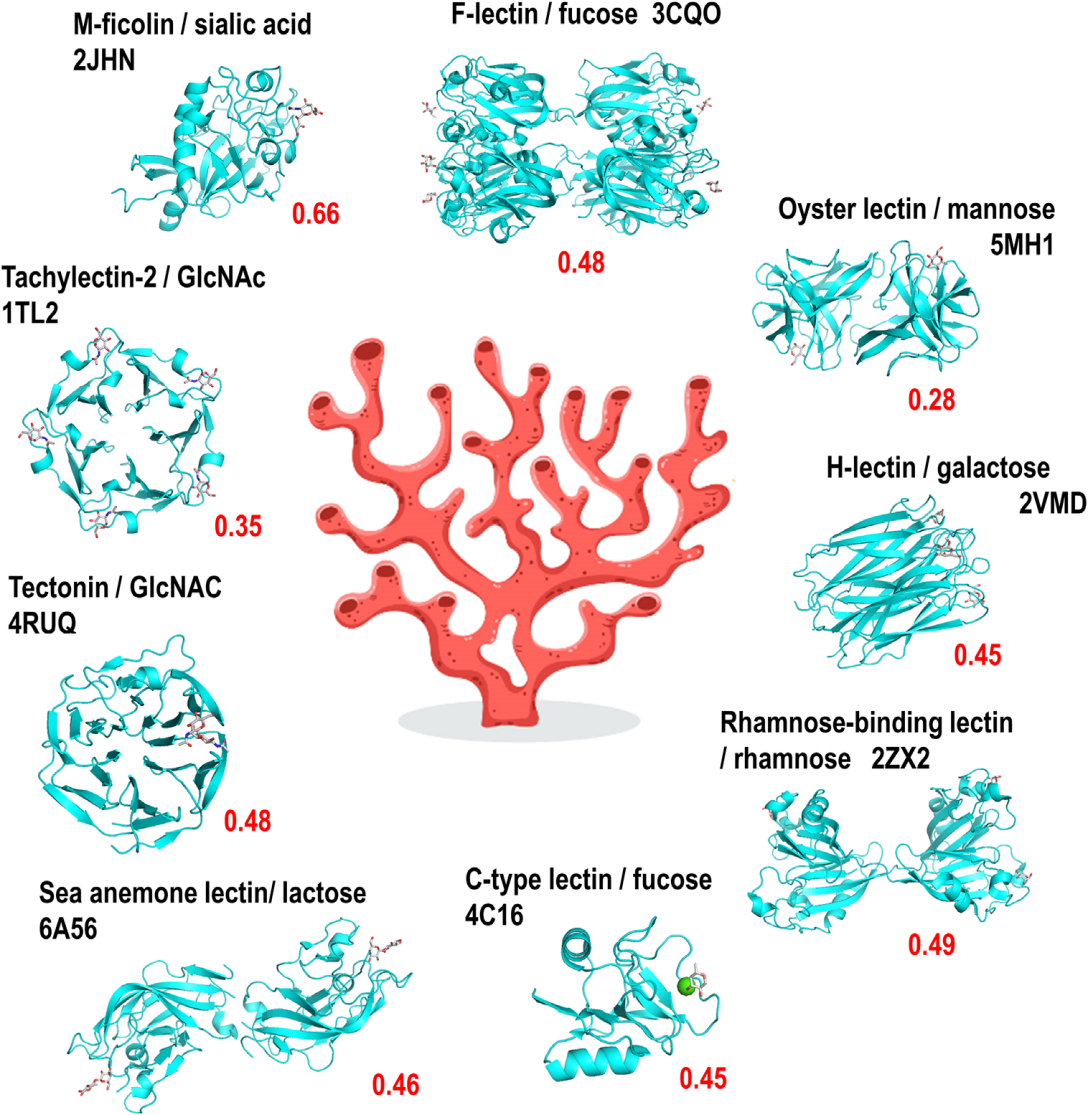
Part of the predicted lectome of corals. Only lectin classes with strong score prediction and conserved binding sites have been selected and represented using Pymol (https://pymol.org/) from selected structures from other origins available in the Protein Data Bank. The scores (red) are calculated for the soft coral *Dendronephthya gigantea*. The hand-written image of coral has been designed by Freepik.com.

As an example, Figure 3 displays a selection of lectins among the 24 predicted to occur in the genome of the soft coral *Dendronephthya gigantea* described in Supplementary Table S5. The prediction also includes widely distributed lectins, such as calnexin, calreticulin and malectin, involved in the quality control during the biosynthesis of glycoproteins. Otherwise, lectins with high score and well conserved carbohydrate binding sites, spread over nine different classes, corresponding to proteins classically involved in innate immunity (Figure 3). It should be noted that only 50% of them are correctly annotated. Some of the identified classes are widely distributed in the animal kingdom, such as ficolins or C-type lectins. F-lectins consist of assembly of fucose-binding domains present in animals and in bacteria (26). On the other hand, some classes are specific to invertebrates, such as oyster lectins or sea anemone lectins and were identified only recently (35–37).

**Figure 4.**
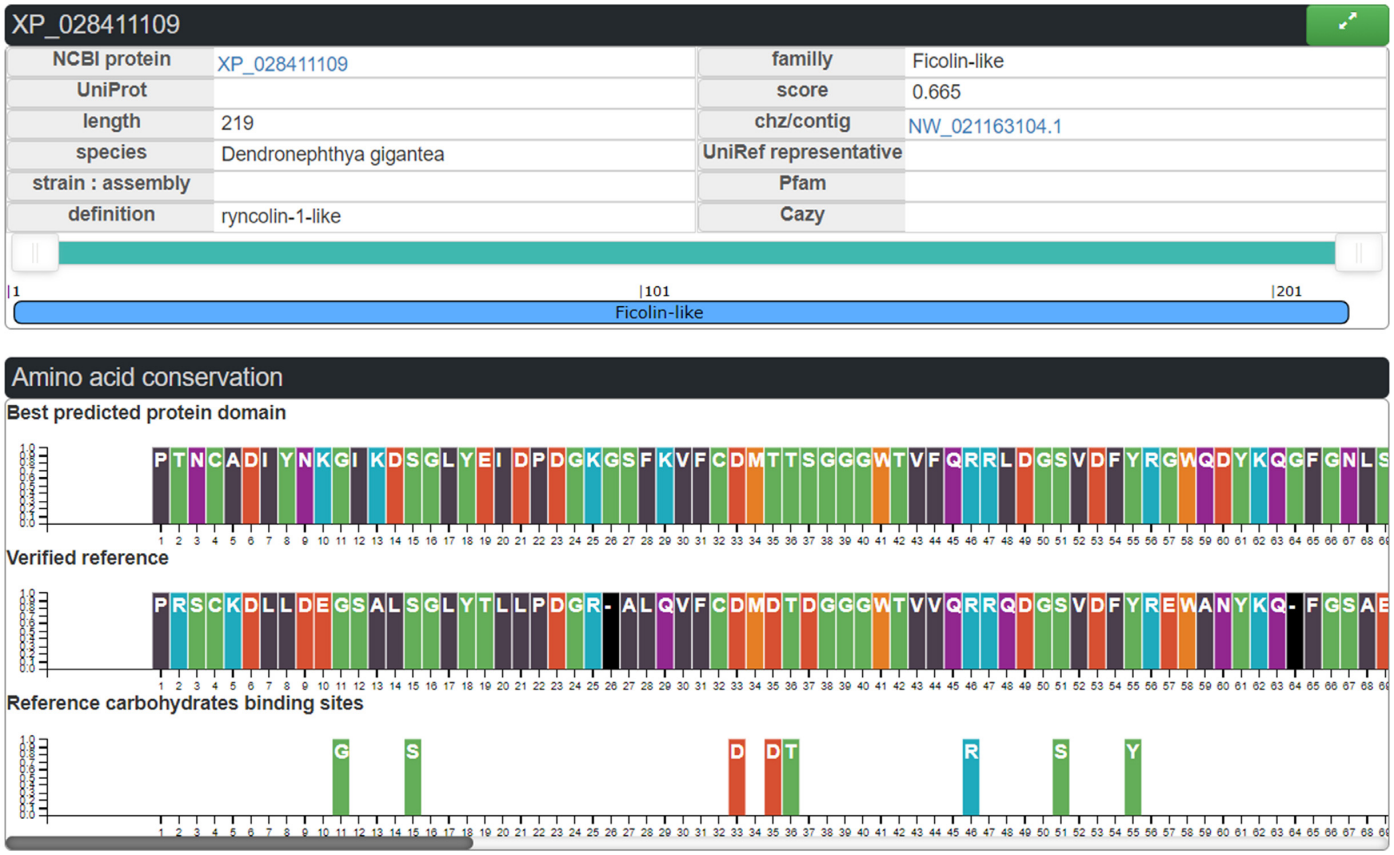
Ficolin-like candidate lectin as represented in LectomeXplore, from the species *Dendronephthya gigantea*. Top: Panel with the main information on the protein. Middle: Panel with the lectin domain aligned with the reference consensus sequence. Bottom: the amino acids involved in glycan-binding in the reference PDB structure.

The LectomeXplore interface gives access to the identification of predicted lectins. For each predicted sequence, the alignment with the reference consensus sequence (generated by HMMER) of the class is displayed when clicking on the arrow in the top right corner of the summary box as shown in Figure 4. This view brings out the quality of the sequence similarity and highlights the amino acids involved in binding sugars below the alignment.

In the analysis of the *Dendronephthya gigantea* lectome, the ficolin-1 class is predicted with a strong score of 0.665. Several proteins are predicted to belong to this lectin class, some of them being annotated as ficolin-2, and others as ryncolin-1-like (Supplementary Table S5), due to their similarity to these snake venom proteins. M-Ficolins bind to acetylated sialic acid on pathogen-associated molecular patterns (PAMPs) (38). The very high conservation in the binding site in the coral ‘ryncolin-1-like’ protein, in particular for basic amino acids, indicates that the sialic acid binding function is likely to be maintained in the coral protein. This example illustrates how LectomeXplore can reveal novel carbohydrate-binding activity in organisms.

## DISCUSSION AND CONCLUSION

The gradual increase of structural data in UniLectin3D was key to determining relevant criteria to classify these proteins. In turn, the resulting classification revealed an underlying order that played a critical role in profiling lectins and support their detection in very large amino acid sequence collections. We surmise that our broad screening strategy will lead not only to consolidate the definition of the chosen criteria but also to improve coverage with the potential creation of new classes or families. The latter could arise from the release of new lectin structures in the PDB. If this classification is adopted by the community, it can serve as a basis for a nomenclature. An option would be to make this information more accessible through an ontology and semantic web technologies (39). But first, we aim at contributing to the annotation of putative lectins in protein sequence databases.

The search tool of the LectomeXplore module is versatile. The occurrence of a given lectin class can be searched in all kingdoms. All lectins present in one species can be searched in whole or a part of a kingdom. Furthermore, lectins/adhesins can be searched in pathogenic bacteria to support an scenario of the strategy used by some pathogenic microbes to attach to the host glycome (22). The prediction of the sugar-binding specificity is still the Holy Grail of glycobioinformatics. This goal is hard to reach because one single mutation in the binding site can have dramatic consequences on specificity. However, LectomeXplore can be useful in synthetic glycobiology, i.e. for engineering new carbohydrate specificities, for example by comparing a large number of predicted lectins and analyzing the mutations that occur in the binding pockets.

UniLectin does not yet provide a public automatic genome analysis tool for the identification of the corresponding lectome. Our analysis is currently limited to newly added genomes in UniProt and NCBI. We are working at it, as it represents a further enhancement of the platform. Nonetheless, any group interested in scanning a genome/proteome or a collection thereof, can contact us for lectome prediction in specific genome data. In addition to lectins with known structure, lectins with unknown structures are also in our focus. Future improvement of LectomeXplore will include a prediction using HMM motifs for each lectin family which is expected to refine the results. To optimize the definition of profiles, we also plan to use structure-based as opposed sequence-based alignment tools.

LectomeXplore is updated every six months. UniLectin3D is first updated based on the recently released 3D structures of lectins. Note that this may also entail revising the classification. Then the conserved motifs of each lectin class are re-generated. Finally, at the time of update, the latest releases of the protein datasets NCBI-nr and UniProtKB are screened to identify lectins. To that end, a dedicated pipeline was developed in Python3. Note that the PropLec module also requires updating, as new classes may be suggested with frequently incoming data. The PropLec methodology was also applied on the β-trefoil and will lead to a future module dedicated only to the β-trefoil lectins, and possibly to other tandem repeat lectins such as β-prisms.

## Supplementary Material

gkaa1019_Supplemental_File
