# Supplementary material for: LectomeXplore, an update of UniLectin for the discovery of carbohydrate-binding proteins based on a new lectin classification
Source: Nucleic Acids Res. 2020 Nov 11;49(D1):D1548–54. doi: 10.1093/nar/gkaa1019 (PMC7778903; doi:10.1093/nar/gkaa1019)
Supplement: gkaa1019_Supplemental_File [file gkaa1019_supplemental_file.docx]

LectomeXplore, an update of Unilectin for the discovery of carbohydrate-binding proteins based on a new lectin classification

François Bonnardel  1,2,3, Julien Mariethoz 2,3,4, Serge Pérez 1, Anne Imberty 1* and Frédérique Lisacek  2,3,4*

1. Univ. Grenoble Alpes, CNRS, CERMAV, 38000 Grenoble, France.

2. Swiss Institute of Bioinformatics, CH-1227 Geneva, Switzerland.

3. Computer Science Department, UniGe, CH-1227 Geneva, Switzerland.

4. Section of Biology, UniGe, CH-1205 Geneva, Switzerland.

* To whom correspondence should be addressed. Anne Imberty (anne.imberty@cermav.cnrs.fr, Tel: +33 476 03 76 40, Twitter: @AnneImberty) Frédérique Lisacek (frederique.lisacek@sib.swiss, Tel: +4122 379 01 95)

# Supplementary materials


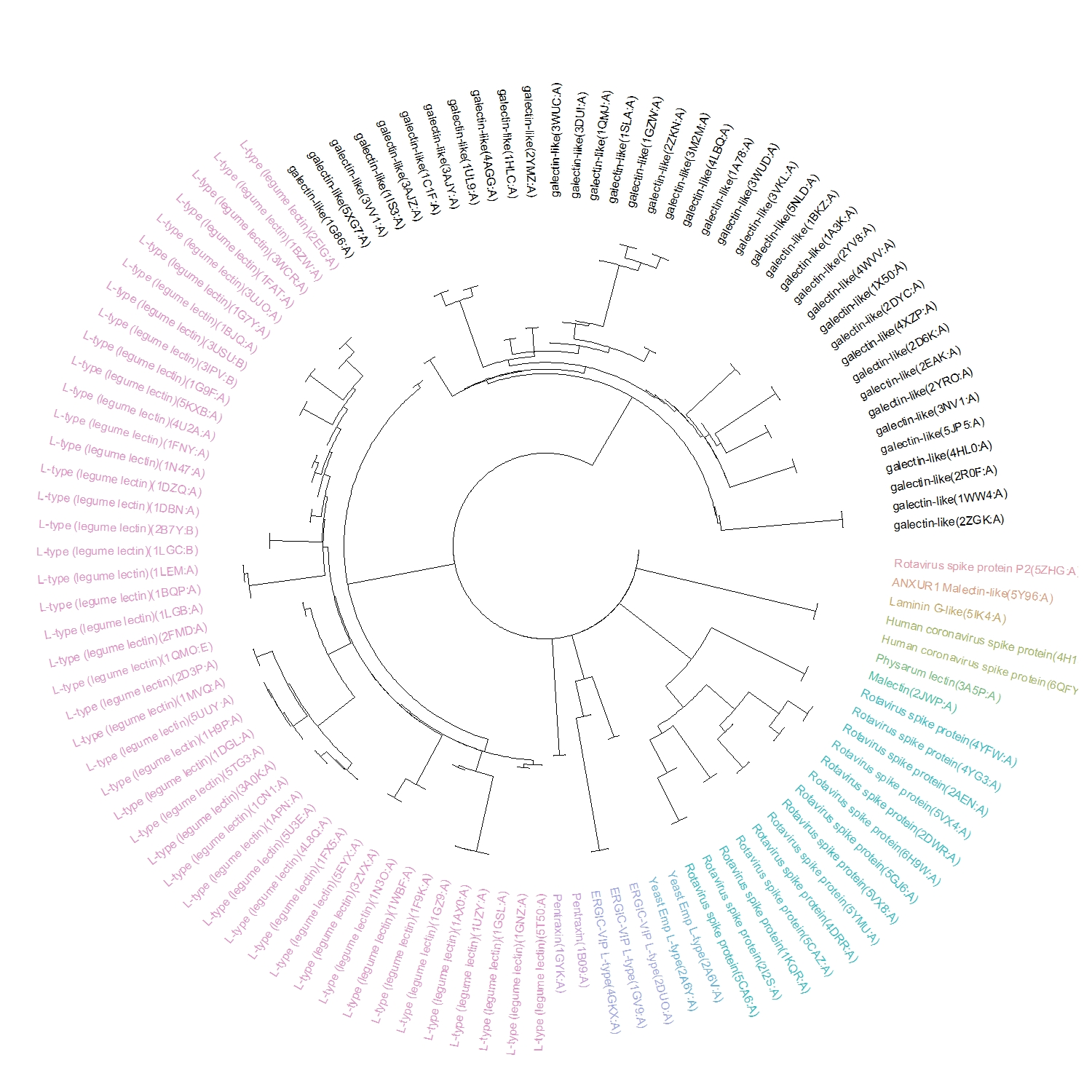


**Supplementary Figure S1:** Hierarchical tree based on the sequence similarity of lectins in UniLectin3D with the fold β-sandwich / ConA-like. The axis goes from 0(inner) to 100%(outer) of sequence similarity. At 20% of identity are defined the lectin classes. The class formed are heterogene and can include a large number of lectins as well as only one isolated lectin.


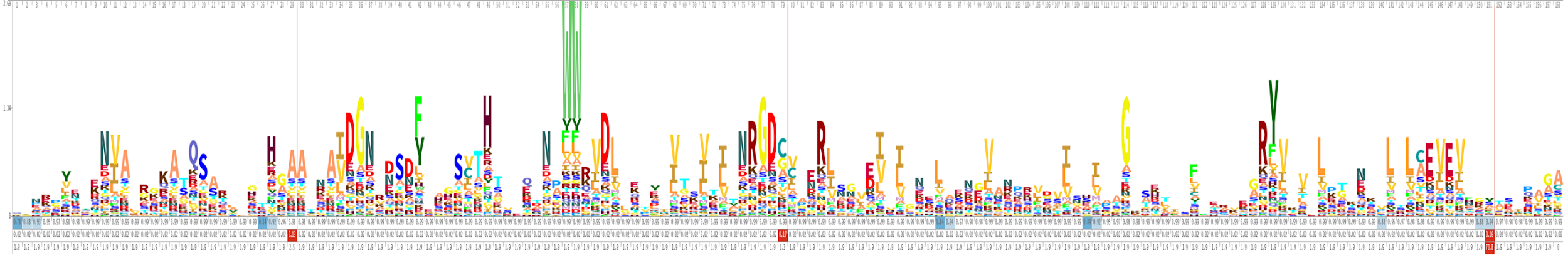


**Supplementary Figure S2:** Conserved motif generated by skylign (skylign.org) based on the lectin f-type domain multiple sequence alignment of the Ficolin-like lectin class

**Supplementary Table S1: Cross-referenced and cited databases and tools**

| **Database name / Tool name** | **URL** | **Information** | **Reference** |
| --- | --- | --- | --- |
| CATH | http://www.cathdb.info/ | Protein domain database with a Structural Classification of Proteins | (Dawson et al., 2017) |
| SCOPe | https://scop.berkeley.edu/ | Protein domain database with a Structural Classification of Proteins | (Chandonia, Fox, & Brenner, 2017) |
| Pfam | https://pfam.xfam.org/ | Protein family databases | (El-Gebali et al., 2019) |
| Lectin Frontier Database (LfDB) | https://acgg.asia/lfdb2/ | Lectin DataBase with quantitative interaction data | (Hirabayashi, Tateno, Shikanai, Aoki-Kinoshita, & Narimatsu, 2015) |
| GlyCosmos | https://glycosmos.org/lectins | Protein entries annotated as lectins in UniProt. | (Yamada et al., 2020) |
| ProCarbDB | http://www.procarbdb.science/procarb/ | X-ray crystal structures of protein-carbohydrate complexes | (Copoiu, Torres, Ascher, Blundell, & Malhotra, 2020) |
| Database of Anti-Glycan Reagents (DAGR) | https://ccr2.cancer.gov/resources/Cbl/Tools/Antibody/About.aspx | database of antibodies and reagent lectins to various carbohydrates | (Sterner, Flanagan, & Gildersleeve, 2016) |
| CAZy CBM | http://www.cazy.org/Carbohydrate-Binding-Modules.html | CBM database, domains within a carbohydrate-active enzyme | (Terrapon, Lombard, Drula, Coutinho, & Henrissat, 2017) |
| Refseq | https://www.ncbi.nlm.nih.gov/refseq/ | well-annotated set of reference sequences | (O’Leary et al., 2016) |
| HMMER | http://hmmer.org/ | biosequence analysis using profile hidden Markov models | (Potter et al., 2018) |
| MUSCLE | https://www.ebi.ac.uk/Tools/msa/muscle/ | Multiple Sequence Alignment | (Edgar, 2004) |
| Glyconnect | https://glyconnect.expasy.org/ | database dedicated to protein glycosylation study | (Alocci et al., 2019) |
| PDBe | https://www.ebi.ac.uk/pdbe/ | 3D structure and structural information | (Mir et al., 2018) |
| RCSB | https://www.rcsb.org/ | 3D structure and structural information | (Burley et al., 2019) |
| SWISS-MODEL templates | https://swissmodel.expasy.org/templates/ | Information on quaternary structure | (Waterhouse et al., 2018) |
| SugarBind | https://sugarbind.expasy.org/ | Specificity of lectins from pathogens | (Mariethoz et al., 2016) |
| SNFG | https://www.ncbi.nlm.nih.gov/glycans/snfg.html | Symbol nomenclature for glycans | (Neelamegham et al., 2019) |
| PubMed | http://www.ncbi.nlm.nih.gov/pubmed | Bibliographic information |  |
| pdb-care | http://www.glycosciences.de/tools/pdb-care/ | Structural check of carbohydrates | (Lütteke & von der Lieth, 2004) |
| Taxonomy | http://www.ncbi.nlm.nih.gov/taxonomy | Organism taxonomy | (Federhen, 2012) |
| UniProtKB | http://www.uniprot.org | Lectin functional annotation | (Bateman, 2019) |
| CFG | http://www.functionalglycomics.org | Experimental data of lectins specificity | (Raman et al., 2006) |

Alocci, D., Mariethoz, J., Gastaldello, A., Gasteiger, E., Karlsson, N. G., Kolarich, D., … Lisacek, F. (2019). GlyConnect: Glycoproteomics Goes Visual, Interactive, and Analytical. *Journal of Proteome Research*, *18*(2), 664–677. https://doi.org/10.1021/acs.jproteome.8b00766

Bateman, A. (2019). UniProt: A worldwide hub of protein knowledge. *Nucleic Acids Research*. https://doi.org/10.1093/nar/gky1049

Burley, S. K., Berman, H. M., Bhikadiya, C., Bi, C., Chen, L., Di Costanzo, L., … Zardecki, C. (2019). RCSB Protein Data Bank: Biological macromolecular structures enabling research and education in fundamental biology, biomedicine, biotechnology and energy. *Nucleic Acids Research*. https://doi.org/10.1093/nar/gky1004

Chandonia, J. M., Fox, N. K., & Brenner, S. E. (2017). SCOPe: Manual Curation and Artifact Removal in the Structural Classification of Proteins – extended Database. *Journal of Molecular Biology*. https://doi.org/10.1016/j.jmb.2016.11.023

Copoiu, L., Torres, P. H. M., Ascher, D. B., Blundell, T. L., & Malhotra, S. (2020). ProCarbDB: A database of carbohydrate-binding proteins. *Nucleic Acids Research*. https://doi.org/10.1093/nar/gkz860

Dawson, N. L., Lewis, T. E., Das, S., Lees, J. G., Lee, D., Ashford, P., … Sillitoe, I. (2017). CATH: An expanded resource to predict protein function through structure and sequence. *Nucleic Acids Research*. https://doi.org/10.1093/nar/gkw1098

Edgar, R. C. (2004). MUSCLE: multiple sequence alignment with high accuracy and high throughput. *Nucleic Acids Research*. https://doi.org/10.1093/nar/gkh340

El-Gebali, S., Mistry, J., Bateman, A., Eddy, S. R., Luciani, A., Potter, S. C., … Finn, R. D. (2019). The Pfam protein families database in 2019. *Nucleic Acids Research*. https://doi.org/10.1093/nar/gky995

Federhen, S. (2012). The NCBI Taxonomy database. *Nucleic Acids Research*. https://doi.org/10.1093/nar/gkr1178

Hirabayashi, J., Tateno, H., Shikanai, T., Aoki-Kinoshita, K. F., & Narimatsu, H. (2015). The lectin frontier database (LfDB), and data generation based on frontal affinity chromatography. *Molecules*. https://doi.org/10.3390/molecules20010951

Lütteke, T., & von der Lieth, C. W. (2004). pdb-care (PDB CArbohydrate REsidue check): A program to support annotation of complex carbohydrate structures in PDB files. *BMC Bioinformatics*, *5*. https://doi.org/10.1186/1471-2105-5-69

Mariethoz, J., Khatib, K., Alocci, D., Campbell, M. P., Karlsson, N. G., Packer, N. H., … Lisacek, F. (2016). SugarBindDB, a resource of glycan-mediated host-pathogen interactions. *Nucleic Acids Research*, *44*(D1), D1243–D1250. https://doi.org/10.1093/nar/gkv1247

Mir, S., Alhroub, Y., Anyango, S., Armstrong, D. R., Berrisford, J. M., Clark, A. R., … Velankar, S. (2018). PDBe: Towards reusable data delivery infrastructure at protein data bank in Europe. *Nucleic Acids Research*, *46*(D1), D486–D492. https://doi.org/10.1093/nar/gkx1070

Neelamegham, S., Aoki-Kinoshita, K., Bolton, E., Frank, M., Lisacek, F., Lütteke, T., … Woods, R. J. (2019). Updates to the Symbol Nomenclature for Glycans guidelines. *Glycobiology*. https://doi.org/10.1093/glycob/cwz045

O’Leary, N. A., Wright, M. W., Brister, J. R., Ciufo, S., Haddad, D., McVeigh, R., … Pruitt, K. D. (2016). Reference sequence (RefSeq) database at NCBI: Current status, taxonomic expansion, and functional annotation. *Nucleic Acids Research*, *44*(D1), D733–D745. https://doi.org/10.1093/nar/gkv1189

Potter, S. C., Luciani, A., Eddy, S. R., Park, Y., Lopez, R., & Finn, R. D. (2018). HMMER web server: 2018 update. *Nucleic Acids Research*. https://doi.org/10.1093/nar/gky448

Raman, R., Venkataraman, M., Ramakrishnan, S., Lang, W., Raguram, S., & Sasisekharan, R. (2006). Advancing glycomics: Implementation strategies at the consortium for functional glycomics. *Glycobiology*. https://doi.org/10.1093/glycob/cwj080

Salentin, S., Schreiber, S., Haupt, V. J., Adasme, M. F., & Schroeder, M. (2015). PLIP: Fully automated protein-ligand interaction profiler. *Nucleic Acids Research*, *43*(W1), W443–W447. https://doi.org/10.1093/nar/gkv315

Sehnal, D., Deshpande, M., Vařeková, R. S., Mir, S., Berka, K., Midlik, A., … Koča, J. (2017). LiteMol suite: interactive web-based visualization of large-scale macromolecular structure data. *Nature Methods*, *14*(12), 1121–1122. https://doi.org/10.1038/nmeth.4499

Sterner, E., Flanagan, N., & Gildersleeve, J. C. (2016). Perspectives on Anti-Glycan Antibodies Gleaned from Development of a Community Resource Database. *ACS Chemical Biology*. https://doi.org/10.1021/acschembio.6b00244

Terrapon, N., Lombard, V., Drula, E., Coutinho, P. M., & Henrissat, B. (2017). The CAZy Database/the Carbohydrate-Active Enzyme (CAZy) Database: Principles and Usage Guidelines. In *A Practical Guide to Using Glycomics Databases*. https://doi.org/10.1007/978-4-431-56454-6_6

Waterhouse, A., Bertoni, M., Bienert, S., Studer, G., Tauriello, G., Gumienny, R., … Schwede, T. (2018). SWISS-MODEL: Homology modelling of protein structures and complexes. *Nucleic Acids Research*. https://doi.org/10.1093/nar/gky427

Yamada, I., Shiota, M., Shinmachi, D., Ono, T., Tsuchiya, S., Hosoda, M., … Aoki-Kinoshita, K. F. (2020). The GlyCosmos Portal: a unified and comprehensive web resource for the glycosciences. *Nature Methods*. https://doi.org/10.1038/s41592-020-0879-8

**Supplementary Table S2:** Full new classification of UniLectin3D with the Fold, the Class defined at 20% of similarity and the Family defined at 70% of similarity.

| Fold | Class | Family |
| --- | --- | --- |
| α-helix triplets / Duffy-like | Plasmodium Erythrocyte binding antigen | FPfEBA-140, PfEBA-175 |
| α/β barrel / TIM | chi-lectin (TCLL) | TCLL |
| α/β barrel / TIM | chi-lectin (TIM) | Hcgp-39, YKL-39, RobpsCRA, Ym1 |
| α/β hairpin / non-globular proline-rich | P-domain of calnexin and reticulin | calreticulin / Entamoeba, calreticulin / mammals, calreticulin / Trypanosoma, calreticulin / NMR, calnexin |
| α/β mixed / C-type lectin-like | C-type lectin | P-selectin, SP-D, dectin-2 / CLEC4N, langerin, SP-A, dectin-1 / CLEC7A, Mincle / CLEC4E, MBP-A / MBL1, DCIR / CLEC4A, DC-SIGN / DC-SIGNR, SRCL / COLEC12, MBP-C / MBL2, collectin-K1 / COLEC11, L-selectin, MMR, MCL / CLEC4D, BDCA-2 / CLEC4C, TC14 / tunicate, CD23, ASGR1, CEL-IV / sea cucumber , E-selectin, DCAR2, DCIR-2, Conglutinin, CEL-I / sea cucumber , SIGNR1 , RSL / rattlesnake, codakine , MBP, tetranectin, MDL-1 / CLEC5A, RegIIIa, Clec4f |
| α/β mixed / LysM domain | LysM-like | Mg1LysM, Ecp6 |
| α/β mixed with b-sheet / Fibrinogen C-ter like | Ficolin-like | L-ficolin,M-ficolin, H-ficolin, intelectin-1 / XEEL, tachilectin-5A, FIBCD |
| α/β mixed with b-sheet / MAR domain | MAR Micronemal protein | TgMIC1-MARR |
| α/β mixed with b-sheet / not classified | Factor H-binding protein | Fhb adhesin |
| α/β mixed with b-sheet / not classified | HOP-OMP adhesins | BabA, SabA |
| α/β mixed with b-sheet / not classified | Mycoplasma adhesins | P110 adhesin |
| α/β OB-fold | Cholera toxin like AB5 | LTB, CTB, CFXB |
| α/β OB-fold | heat labile toxin IIB AB5 | LT-IIb |
| α/β OB-fold | Pertussis toxin AB5 | pertussis toxin |
| α/β OB-fold | Shiga toxin AB5 | SLT-2 / STX-2, SLT-1 / STX-1 |
| α/β OB-fold | Staphylococcal enterotoxin | SEC2 |
| α/β OB-fold | Staphylococcal Superantigen-Like | SSL3, SSL5, SSL11 |
| α/β OB-fold | Thyphoid toxin like AB5 | PltB, SubB, ArtB |
| β-barrel | OAA-like | OAAH / PFA, OAAH |
| β-barrel | P-type lectin | CI-MPR, CD-MPR |
| β-barrel | P-type lectin-like | MRH |
| β-hairpin stack | Toxin repetitive domain | Clostridium repeated domains |
| β-helix | Acinetobacter phage AP22 | AP22 |
| β-helix | E coli bacteriophage | HK620 |
| β-helix | E coli phage CBA120 | CBA120 |
| β-helix | Salmonella bacteriophage | P22 |
| β-helix | Shigella phage Sf6 | Sf6 |
| β-helix | Variable Lymphocyte Receptor | Lamprey VLR |
| β-prism I | Jacalin-like | banana, artocarpin, MornigaM, Frutapin, jacalin, ZG16p, ipomoelin, PPL3, parkia, griffithsin, jacalin AHL,MPA,artocarpin, WGA16, ananas, Calsepa, Orysata, heltuba |
| β-prism I | Natterin-like | Dln1 |
| β-prism I | Oyster lectin | CgDM9CP-1 |
| β-prism I | Vibrio β-prism | VCC cytolysin, RbmC |
| β-prism II | aMan-specific plant lectin | SCA-Man, amaryllis |
| β-prism II | Monocot-lectin like | Mycobacterium smegmatis, LlpA, Remusatia, Colocosia, garlic, SCA-FET, Polygonatum, Pyocin, GNL, narcissus, gastrodiadin, MOL, Crocus vernus, SCA-Man |
| β-prism II | Pyocin | Pyocin |
| β-prism III | Fungal prism lectins | PhoSL |
| β-propeller | AAL-like (PropLec6A) | RSL, BambL, AOL, AFL / FleA, AAL, KozL |
| β-propeller | BPL and CVL like (PropLec7C) | CVL, BPL |
| β-propeller | neuraminidase-like | Measles virus |
| β-propeller | PLL-like (PropLec7A) | PHL, PLL-like, PLL2, PLL, PLL3 |
| β-propeller | PVL-like (PropLec7B) | PVL, PAL, AAL2 |
| β-propeller | Tachylectin-2-like (PropLec5A) | Tachylectin-2-like, tachylectin-2 |
| β-propeller | Tectonin (PropLec6B) | tectonin-2, fish-egg lectin |
| β-sandwich / 2 calcium lectin | 2 calcium lectin | LecB / PA-IIL, RSIIL, BC2L-A, CV-IIL, BC2L-C-Cter |
| β-sandwich / ConA-like | ANXUR1 Malectin-like | Malectin |
| β-sandwich / ConA-like | Coronavirus spike protein | MERS-CoV, HCoV, BCoV |
| β-sandwich / ConA-like | ERGIC-VIP L-type | VIP36, ERGIC-53 |
| β-sandwich / ConA-like | galectin-like | galectin-13, galectin-4, galectin-8, agrocybe ACG, Sponge galectin, galectin-5, galectin-9, galectin-9, coprinus galectin-2, chicken grifin, toad galectin, Nematode galectin-9, galectin-1, galectin-3, galectin-10 (Charcot-Leyden crystal protein), congerin, galectin-7, coprinus galectin-3, galectin-2, Nematode galectin-6, xgalectin-Ib, xgalectin-Va, CG-2, chicken CG1-A, chicken galectin |
| β-sandwich / ConA-like | L-type (legume lectin) | platypodium and related, ConA and related, Butea (Erythrina) monosperma, Spatholobus parviflorus, UEA-1, VML, EcorL and ECL, PNA, GSL-IV, favin, LOL, pea, lens, WFA, WBA-I, PHA-E, PHA-L, BFL, Dolichos biflorus DBL and DB59, SBA, Dolichos biflorus DBL and DB58, UEA-2, RPbAI, Lotus, TBL, GSL-I, FRIL, BMA, WBA-II, VVL-B4, MAL |
| β-sandwich / ConA-like | L-type lectin | Dolichos biflorus DBL and DB58, lima bean LBL, ConA and related, Ulex europaeus UEA-1 |
| β-sandwich / ConA-like | Laminin G-like | Laminin-1 |
| β-sandwich / ConA-like | Malectin | Malectin |
| β-sandwich / ConA-like | Pentraxin | limulus polyphemus, serum amyloid P component, Mesocricetus auratus, C-reactive protein |
| β-sandwich / ConA-like | Physarum lectin | physarum |
| β-sandwich / ConA-like | Rotavirus spike protein | Bovine-human rotavirus [P11], Human rotavirus P[8], P[4], Human rotavirus P[6], P[19], Porcine rotavirus P[7], P[3], Human rotavirus P[14], P[9] |
| β-sandwich / ConA-like | Rotavirus spike protein P2 | Human rotavirus P[2] |
| β-sandwich / ConA-like | Yeast Emp L-type | Emp46p, Emp47p |
| b-sandwich / CUB-like | L-rhamnose binding lectin | CSL3, latrophilin-1, SUL-I |
| β-sandwich / cyanovirin-like | cyanovirin-like | TbCVNH and NcCVNH chimera, CVNH, Cyt-CVNH, NcCVNH, Microvirin, CrCVNH, MoCVNH, TbCVNH, GzCVNH |
| β-sandwich / cyanovirin-like | Microvirin CVN-like | Microvirin |
| β-sandwich / cytolysin-like | Fungal fruit body lectin | SRL, BEL and XCL, ABL |
| β-sandwich / cytolysin-like | Oomycete cytolysin | NLP cytolysin |
| β-sandwich / Galactose-binding domain-like | 1 Calcium lectin | LecA / PA-IL, PIIA |
| β-sandwich / Galactose-binding domain-like | CBM67-like | POL |
| β-sandwich / Galactose-binding domain-like | F-type lectin | Lectinolysin, AAA, bass lectin |
| β-sandwich / Galactose-binding domain-like | H-type lectin | HPA, HAA, discoidin II, discoidin I, SLL-2 |
| β-sandwich / Galactose-binding domain-like | Sea anemon lectin | AJLec |
| β-sandwich / Ig-like | I-type lectin | MAG, sialoadhesin, Siglec-7, Siglec-5, Siglec-like PILR, Siglec-like CD33 , Siglec-8 |
| β-sandwich / Ig-like | Ig-like | Fip-Fve |
| β-sandwich / Ig-like | serine-rich repeat adhesin | SrpA, GspB, HSA, HSA_like_NCTC10712, HSA_like_SK678, GspB_like_SK150 |
| β-sandwich / PA14 adhesin | PA14 RTX | MhPA14, MpAFP |
| β-sandwich / PA14 adhesin | PA14 yeast adhesin | Flo1 and Flo6, Epa1A, Epa6A, Epa9A, Flo1, Flo2 |
| β-sandwich / pili and adhesins | bacterial adhesin (CfaE) | CfaE |
| β-sandwich / pili and adhesins | bacterial adhesin (FaeG) | FaeG |
| β-sandwich / pili and adhesins | bacterial adhesin (FedF) | FedF / F18 |
| β-sandwich / pili and adhesins | bacterial adhesin (FimH-FlmH) | FmlH, FlmH, FimH |
| β-sandwich / pili and adhesins | bacterial adhesin (GafD) | GafD / F17-G |
| β-sandwich / pili and adhesins | bacterial adhesin (PapG) | PapG |
| β-sandwich / pili and adhesins | bacterial adhesin (PsaA) | MyfA, PsaA |
| β-sandwich / pili and adhesins | bacterial adhesin (UclD) | UclD / F17-like |
| β-sandwich / TNF-like | TNFa-like | Bc2L-C-Nter |
| β-sandwich / viral coat and capsid protein | Bluetongue virus coat protein | Bluetongue virus |
| β-sandwich / viral coat and capsid protein | Equine Rhinitis A Virus Coat Protein | Equine Rhinitis A virus |
| β-sandwich / viral coat and capsid protein | FMDV receptor complex | Norovirus GII 1, GII 10, GII 12, GII 18, foot-and-mouth disease virus, Norovirus GII 4, Norovirus GI 7, Norovirus GII 1, GII 10, GII 12, GII 17, coxsackievirus A24, Norovirus GII 1, GII 10, GII 12, GII 20, Norovirus GI 1, Murine norovirus, Norovirus GI 2, Norovirus GII 9, Norovirus GII 13, GII 21, Norovirus GI 8, Norovirus GII 2, Norovirus GII 3, Norovirus GII 1, GII 10, GII 12, GII 19, bovine norovirus |
| β-sandwich / viral coat and capsid protein | Polyomavirus capsid protein | Murine polyomavirus, Human JC polyomavirus,BKPyV,SV 40, Merkel Cell Polyomavirus, KIPyV, WUPyV, Human JC polyomavirus,BKPyV,SV 41, Human JC polyomavirus,BKPyV,SV 42, HPyV9, LPyV, TSPyV |
| β-sandwich / viral protein domain | hemagglutinin-esterase | bovine coronavirus |
| β-sandwich / viral protein domain | Influenza hemagglutinin | influenza virus |
| β-sandwich / virus globular domain | Fiber knob | human adenovirus, mastadenovirus G, adenovirus, canine adenovirus |
| β-sandwich / virus globular domain | Fiber-knob parvovirus | dependoparvovirus A |
| β-sandwich / virus globular domain | Phage binding domain | RBB phage |
| β-sandwich / virus globular domain | Turkey siadenovirus A | Porcine adenovirus and related |
| β-trefoil | Amaranthin-like | amaranthin |
| β-trefoil | Boletus and Laetiporus β-trefoil lectin | BEL, LSLa |
| β-trefoil | Clitocybe lectin-like | CNL |
| β-trefoil | Clostridial toxin | BoNT/A, BoNT/C, TeNT, BoNT/D, BoNT/G, BoNT/B, BoNT/F |
| β-trefoil | Coprinus β-trefoil lectin | CCL2 |
| β-trefoil | Cys-rich man-receptor | Cys-rich domain man-receptor |
| β-trefoil | Earthworm lectin | earthworm EW29 |
| β-trefoil | Fungi and Clostridium β-trefoil lectin | (HA1) HA-33/A, (HA3) HA70/A, RSA, (HA1) HA-33/D and C, Macrolepiota, MOA, (HA3) HA70/C, PSL |
| β-trefoil | Mussel lectin | mussel lectin, synthetic mussel lectin |
| β-trefoil | R-type lectin | VAA |
| β-trefoil | Ricin-like | Momordica lectin, SNA-II, Trichosanthes lectin, sea cucumber CEL-III, actinohivin, VAA, ricin V, Abrus agglutinin,abrin-a, Vibrio vulnificus, TKL-1 |
| β-trefoil | Sclerotinia lectin like | Sclerotinia |
| peptide | Trefoil Factor | TFF3 |
| small protein / APPLE domain | SML2 Micronemal protein | SML-2, TgMIC4 |
| small protein / disulfide rich | Cyanobacterial scytovirin | scytovirin |
| small protein / Invertebrate chitin-binding protein | Invertebrate chitin-binding protein | tachycitin |
| small protein / Knottin | Ginkbilobin | Ginkbilobin-2 |
| small protein / Knottin | Ginkbilobin-like | Coprinus Y3 |
| small protein / Knottin | hevein-like | Pokeweed lectin, WGA, Hevein, UDA, Amaranthus antimicrobial peptide |
| small protein / Knottin | Lyophyllum ginkbilobin-like | LDL |
| small protein / Knottin | Spider toxin | selenocosmia huwena lectin-I |

**Supplementary Table S3:**  Pfam entries sharing at least one PDB cross-reference with a lectin class. The highlighted lines in red contain Pfams not well described in the Pfam database.

| **Representative shared PDB structure** | **lectin class** | **lectin fold** | **pfam** | **pfam name** |
| --- | --- | --- | --- | --- |
| 4GF2:A | Plasmodium Erythrocyte binding antigen | α-helix triplets / Duffy-like | [PF05424](https://pfam.xfam.org/family/PF05424) | Duffy_binding |
| 4B16:A | chi-lectin (TCLL) | α/β barrel / TIM | [PF00704](https://pfam.xfam.org/family/PF00704) | Glyco_hydro_18 |
| 1HJX:A | chi-lectin (TIM) | α/β barrel / TIM | [PF00704](https://pfam.xfam.org/family/PF00704) | Glyco_hydro_18 |
| 1JHN:A | P-domain of calnexin and reticulin | α/β hairpin / non-globular proline-rich | [PF00262](https://pfam.xfam.org/family/PF00262) | Calreticulin |
| 1G1T:A | C-type lectin | α/β mixed / C-type lectin-like | [PF00008](https://pfam.xfam.org/family/PF00008) | EGF |
| 1DV8:A | C-type lectin | α/β mixed / C-type lectin-like | [PF00059](https://pfam.xfam.org/family/PF00059) | Lectin C |
| 4CSY:A | C-type lectin | α/β mixed / C-type lectin-like | [PF00084](https://pfam.xfam.org/family/PF00084) | Sushi |
| 4M18:A | C-type lectin | α/β mixed / C-type lectin-like | [PF09006](https://pfam.xfam.org/family/PF09006) | Surfac_D-trimer |
| 4B9H:A | LysM-like | α/β mixed / LysM domain | [PF01476](https://pfam.xfam.org/family/PF01476) | LysM |
| 4M7F:A | Ficolin-like | α/β mixed with β-sheet / Fibrinogen C-ter like | [PF00147](https://pfam.xfam.org/family/PF00147) | Fibrinogen_C |
| 3F53:A | MAR Micronemal protein | α/β mixed with β-sheet / MAR domain | [PF10564](https://pfam.xfam.org/family/PF10564) | MAR_sialic_bdg |
| 4ZH7:A | HOP-OMP adhesins | α/β mixed with β-sheet / not classified | [PF18304](https://pfam.xfam.org/family/PF18304) | SabA_adhesion |
| 6R41:A | Mycoplasma adhesins | α/β mixed with β-sheet / not classified | [PF05220](https://pfam.xfam.org/family/PF05220) | MgpC |
| 1EFI:D | Cholera toxin like AB5 | α/β OB-fold | [PF01376](https://pfam.xfam.org/family/PF01376) | Enterotoxin_b |
| 1TII:D | heat labile toxin IIB AB5 | α/β OB-fold | [PF06453](https://pfam.xfam.org/family/PF06453) | LT-IIB |
| 1PTO:D | Pertussis toxin AB5 | α/β OB-fold | [PF09275](https://pfam.xfam.org/family/PF09275) | Pertus-S4-tox |
| 2C5C:A | Shiga toxin AB5 | α/β OB-fold | [PF02258](https://pfam.xfam.org/family/PF02258) | SLT_beta |
| 1SE3:A | Staphylococcal enterotoxin | α/β OB-fold | [PF01123](https://pfam.xfam.org/family/PF01123) | Stap_Strp_toxin |
| 1SE3:A | Staphylococcal enterotoxin | α/β OB-fold | [PF02876](https://pfam.xfam.org/family/PF02876) | Stap_Strp_tox_C |
| 2RDG:A | Staphylococcal Superantigen-Like | α/β OB-fold | [PF02876](https://pfam.xfam.org/family/PF02876) | Stap_Strp_tox_C |
| 2RDG:A | Staphylococcal Superantigen-Like | α/β OB-fold | [PF09199](https://pfam.xfam.org/family/PF09199) | SSL_OB |
| 4K6L:A | Thyphoid toxin like AB5 | α/β OB-fold | [PF02918](https://pfam.xfam.org/family/PF02918) | Pertussis_S2S3 |
| 3S5X:A | OAA-like | β-barrel | [PF17882](https://pfam.xfam.org/family/PF17882) | SBD |
| 1SZ0:A | P-type lectin | β-barrel | [PF00878](https://pfam.xfam.org/family/PF00878) | CIMR |
| 1M6P:A | P-type lectin | β-barrel | [PF02157](https://pfam.xfam.org/family/PF02157) | Man-6-P_recep |
| 3AIH:A | P-type lectin-like | β-barrel | [PF07915](https://pfam.xfam.org/family/PF07915) | PRKCSH |
| 2G7C:A | Toxin repetitive domain | β-hairpin stack | [PF01473](https://pfam.xfam.org/family/PF01473) | CW_binding_1 |
| 4YEJ:A | E coli bacteriophage | β-helix | [PF18781](https://pfam.xfam.org/family/PF18781) | Phage_spike_2 |
| 4OJ6:A | E coli phage CBA120 | β-helix | [PF18668](https://pfam.xfam.org/family/PF18668) | Tail_spike_N |
| 1CLW:A | Salmonella bacteriophage | β-helix | [PF09251](https://pfam.xfam.org/family/PF09251) | PhageP22-tail |
| 4URR:A | Shigella phage Sf6 | β-helix | [PF12708](https://pfam.xfam.org/family/PF12708) | Pectate_lyase_3 |
| 3G39:A | Variable Lymphocyte Receptor | β-helix | [PF01462](https://pfam.xfam.org/family/PF01462) | LRRNT |
| 2R9U:A | Variable Lymphocyte Receptor | β-helix | [PF11921](https://pfam.xfam.org/family/PF11921) | DUF3439 |
| 2R9U:A | Variable Lymphocyte Receptor | β-helix | [PF13855](https://pfam.xfam.org/family/PF13855) | LRR_8 |
| 2GTY:A | Jacalin-like | β-prism I | [PF01419](https://pfam.xfam.org/family/PF01419) | Jacalin |
| 4ZNO:A | Natterin-like | β-prism I | [PF03318](https://pfam.xfam.org/family/PF03318) | ETX_MTX2 |
| 5ID8:A | Oyster lectin | β-prism I | [PF11901](https://pfam.xfam.org/family/PF11901) | DUF3421 |
| 1XEZ:A | Vibrio β-prism | β-prism I | [PF07968](https://pfam.xfam.org/family/PF07968) | Leukocidin |
| 1XEZ:A | Vibrio β-prism | β-prism I | [PF12563](https://pfam.xfam.org/family/PF12563) | Hemolysin_N |
| 5V6F:A | Vibrio β-prism | β-prism I | [PF16458](https://pfam.xfam.org/family/PF16458) | Beta-prism_lec |
| 3MEZ:A | Monocot-lectin like | β-prism II | [PF01453](https://pfam.xfam.org/family/PF01453) | B_lectin |
| 3MFB:A | Pyocin | β-prism II | [PF06958](https://pfam.xfam.org/family/PF06958) | Pyocin_S |
| 5AJC:A | AAL-like (PropLec6A) | β-propeller | [PF07938](https://pfam.xfam.org/family/PF07938) | Fungal_lectin |
| 2RKC:A | neuraminidase-like | β-propeller | [PF00423](https://pfam.xfam.org/family/PF00423) | HN |
| 6FHX:A | PLL-like (PropLec7A) | β-propeller | [PF03984](https://pfam.xfam.org/family/PF03984) | DUF346 |
| 4UP4:A | PVL-like (PropLec7B) | β-propeller | [PF13517](https://pfam.xfam.org/family/PF13517) | VCBS |
| 1TL2:A | Tachylectin-2-like (PropLec5A) | β-propeller | [PF14517](https://pfam.xfam.org/family/PF14517) | Tachylectin |
| 4RUQ:A | Tectonin (PropLec6B) | β-propeller | [PF06462](https://pfam.xfam.org/family/PF06462) | Hyd_WA |
| 2WRA:A | 2 calcium lectin | β-sandwich / 2 calcium lectin | [PF07472](https://pfam.xfam.org/family/PF07472) | PA-IIL |
| 5Y96:A | ANXUR1 Malectin-like | β-sandwich / ConA-like | [PF12819](https://pfam.xfam.org/family/PF12819) | Malectin_like |
| 6NZK:A | Coronavirus spike protein | β-sandwich / ConA-like | [PF01601](https://pfam.xfam.org/family/PF01601) | Corona_S2 |
| 6NZK:A | Coronavirus spike protein | β-sandwich / ConA-like | [PF09408](https://pfam.xfam.org/family/PF09408) | Spike_rec_bind |
| 6NZK:A | Coronavirus spike protein | β-sandwich / ConA-like | [PF16451](https://pfam.xfam.org/family/PF16451) | Spike_NTD |
| 1GV9:A | ERGIC-VIP L-type | β-sandwich / ConA-like | [PF03388](https://pfam.xfam.org/family/PF03388) | Lectin_leg-like |
| 3DUI:A | galectin-like | β-sandwich / ConA-like | [PF00337](https://pfam.xfam.org/family/PF00337) | Gal-bind_lectin |
| 5T5O:A | L-type (legume lectin) | β-sandwich / ConA-like | [PF00139](https://pfam.xfam.org/family/PF00139) | Lectin_legB |
| 5IK5:A | Laminin G-like | β-sandwich / ConA-like | [PF00054](https://pfam.xfam.org/family/PF00054) | Laminin_G_1 |
| 5IK5:A | Laminin G-like | β-sandwich / ConA-like | [PF02210](https://pfam.xfam.org/family/PF02210) | Laminin_G_2 |
| 2JWP:A | Malectin | β-sandwich / ConA-like | [PF11721](https://pfam.xfam.org/family/PF11721) | Malectin |
| 1B09:A | Pentraxin | β-sandwich / ConA-like | [PF00354](https://pfam.xfam.org/family/PF00354) | Pentaxin |
| 3A5P:A | Physarum lectin | β-sandwich / ConA-like | [PF18239](https://pfam.xfam.org/family/PF18239) | HA1 |
| 2I2S:A | Rotavirus spike protein | β-sandwich / ConA-like | [PF00426](https://pfam.xfam.org/family/PF00426) | VP4_haemagglut |
| 5ZHO:A | Rotavirus spike protein P2 | β-sandwich / ConA-like | [PF00426](https://pfam.xfam.org/family/PF00426) | VP4_haemagglut |
| 2A6Y:A | Yeast Emp L-type | β-sandwich / ConA-like | [PF03388](https://pfam.xfam.org/family/PF03388) | Lectin_leg-like |
| 2ZX0:A | L-rhamnose binding lectin | β-sandwich / CUB-like | [PF02140](https://pfam.xfam.org/family/PF02140) | Gal_Lectin |
| 2L9Y:A | cyanovirin-like | β-sandwich / cyanovirin-like | [PF01476](https://pfam.xfam.org/family/PF01476) | LysM |
| 4J4D:A | cyanovirin-like | β-sandwich / cyanovirin-like | [PF08881](https://pfam.xfam.org/family/PF08881) | CVNH |
| 1ZHS:A | Microvirin CVN-like | β-sandwich / cyanovirin-like | [PF12151](https://pfam.xfam.org/family/PF12151) | MVL |
| 1Y2U:A | Fungal fruit body lectin | β-sandwich / cytolysin-like | [PF07367](https://pfam.xfam.org/family/PF07367) | FB_lectin |
| 5NNW:A | Oomycete cytolysin | β-sandwich / cytolysin-like | [PF05630](https://pfam.xfam.org/family/PF05630) | NPP1 |
| 4LJH:A | 1 Calcium lectin | β-sandwich / Galactose-binding domain-like | [PF07828](https://pfam.xfam.org/family/PF07828) | PA-IL |
| 1K12:A | F-type lectin | β-sandwich / Galactose-binding domain-like | [PF00754](https://pfam.xfam.org/family/PF00754) | F5_F8_type_C |
| 2W94:A | H-type lectin | β-sandwich / Galactose-binding domain-like | [PF00754](https://pfam.xfam.org/family/PF00754) | F5_F8_type_C |
| 2W94:A | H-type lectin | β-sandwich / Galactose-binding domain-like | [PF09458](https://pfam.xfam.org/family/PF09458) | H_lectin |
| 5J06:A | I-type lectin | β-sandwich / Ig-like | [PF00047](https://pfam.xfam.org/family/PF00047) | ig |
| 5J06:A | I-type lectin | β-sandwich / Ig-like | [PF07686](https://pfam.xfam.org/family/PF07686) | V-set |
| 5LFV:A | I-type lectin | β-sandwich / Ig-like | [PF08205](https://pfam.xfam.org/family/PF08205) | C2-set_2 |
| 5LFV:A | I-type lectin | β-sandwich / Ig-like | [PF13927](https://pfam.xfam.org/family/PF13927) | Ig_3 |
| 1OSY:A | Ig-like | β-sandwich / Ig-like | [PF09259](https://pfam.xfam.org/family/PF09259) | Fve |
| 5J6Y:A | PA14 RTX | β-sandwich / PA14 adhesin | [PF07691](https://pfam.xfam.org/family/PF07691) | PA14 |
| 2XJV:A | PA14 yeast adhesin | β-sandwich / PA14 adhesin | [PF07691](https://pfam.xfam.org/family/PF07691) | PA14 |
| 4COV:A | PA14 yeast adhesin | β-sandwich / PA14 adhesin | [PF10528](https://pfam.xfam.org/family/PF10528) | GLEYA |
| 2HB0:A | bacterial adhesin (CfaE) | β-sandwich / pili and adhesins | [PF07434](https://pfam.xfam.org/family/PF07434) | CblD |
| 1KLF:B | bacterial adhesin (FimH-FlmH) | β-sandwich / pili and adhesins | [PF00419](https://pfam.xfam.org/family/PF00419) | Fimbrial |
| 4AV5:A | bacterial adhesin (FimH-FlmH) | β-sandwich / pili and adhesins | [PF09160](https://pfam.xfam.org/family/PF09160) | FimH_man-bind |
| 2BS8:A | bacterial adhesin (GafD) | β-sandwich / pili and adhesins | [PF09222](https://pfam.xfam.org/family/PF09222) | Fim-adh_lectin |
| 4Z3H:A | bacterial adhesin (PapG) | β-sandwich / pili and adhesins | [PF03627](https://pfam.xfam.org/family/PF03627) | PapG_N |
| 3IYK:G | Bluetongue virus coat protein | β-sandwich / viral coat and capsid protein | [PF00898](https://pfam.xfam.org/family/PF00898) | Orbi_VP2 |
| 4Q4V:2 | FMDV receptor complex | β-sandwich / viral coat and capsid protein | [PF00073](https://pfam.xfam.org/family/PF00073) | Rhv |
| 4RPB:A | FMDV receptor complex | β-sandwich / viral coat and capsid protein | [PF00915](https://pfam.xfam.org/family/PF00915) | Calici_coat |
| 3LQ6:A | FMDV receptor complex | β-sandwich / viral coat and capsid protein | [PF08435](https://pfam.xfam.org/family/PF08435) | Calici_coat_C |
| 4MBX:A | Polyomavirus capsid protein | β-sandwich / viral coat and capsid protein | [PF00718](https://pfam.xfam.org/family/PF00718) | Polyoma_coat |
| 3CL5:A | hemagglutinin-esterase | β-sandwich / viral protein domain | [PF02710](https://pfam.xfam.org/family/PF02710) | Hema_HEFG |
| 3CL5:A | hemagglutinin-esterase | β-sandwich / viral protein domain | [PF03996](https://pfam.xfam.org/family/PF03996) | Hema_esterase |
| 4BSC:B | Influenza hemagglutinin | β-sandwich / viral protein domain | [PF00509](https://pfam.xfam.org/family/PF00509) | Hemagglutinin |
| 5EGC:A | Fiber-knob parvovirus | β-sandwich / virus globular domain | [PF00740](https://pfam.xfam.org/family/PF00740) | Parvo_coat |
| 6FJO:A | Fiber knob | β-sandwich / virus globular domain | [PF00541](https://pfam.xfam.org/family/PF00541) | Adeno_knob |
| 2WSU:A | Turkey siadenovirus A | β-sandwich / virus globular domain | [PF00337](https://pfam.xfam.org/family/PF00337) | Gal-bind_lectin |
| 1JLX:A | Amaranthin-like | β-trefoil | [PF07468](https://pfam.xfam.org/family/PF07468) | Agglutinin |
| 1W3G:A | Boletus and Laetiporus β-trefoil lectin | β-trefoil | [PF03318](https://pfam.xfam.org/family/PF03318) | ETX_MTX2 |
| 3NBC:A | Clitocybe lectin-like | β-trefoil | [PF14200](https://pfam.xfam.org/family/PF14200) | RicinB_lectin_2 |
| 3BTA:A | Clostridial toxin | β-trefoil | [PF01742](https://pfam.xfam.org/family/PF01742) | Peptidase_M27 |
| 2VU9:A | Clostridial toxin | β-trefoil | [PF07951](https://pfam.xfam.org/family/PF07951) | Toxin_R_bind_C |
| 3BTA:A | Clostridial toxin | β-trefoil | [PF07952](https://pfam.xfam.org/family/PF07952) | Toxin_trans |
| 2VU9:A | Clostridial toxin | β-trefoil | [PF07953](https://pfam.xfam.org/family/PF07953) | Toxin_R_bind_N |
| 1FWU:A | Cys-rich man-receptor | β-trefoil | [PF00652](https://pfam.xfam.org/family/PF00652) | Ricin_B_lectin |
| 2DRY:A | Earthworm lectin | β-trefoil | [PF00652](https://pfam.xfam.org/family/PF00652) | Ricin_B_lectin |
| 4LO5:A | Fungi and Clostridium β-trefoil lectin | β-trefoil | [PF03505](https://pfam.xfam.org/family/PF03505) | Clenterotox |
| 1YBI:A | Fungi and Clostridium β-trefoil lectin | β-trefoil | [PF05588](https://pfam.xfam.org/family/PF05588) | Botulinum_HA-17 |
| 3EF2:A | Fungi and Clostridium β-trefoil lectin | β-trefoil | [PF14200](https://pfam.xfam.org/family/PF14200) | RicinB_lectin_2 |
| 5XG5:A | Mussel lectin | β-trefoil | [PF14200](https://pfam.xfam.org/family/PF14200) | RicinB_lectin_2 |
| 2Q3N:B | Ricin-like | β-trefoil | [PF00652](https://pfam.xfam.org/family/PF00652) | Ricin_B_lectin |
| 2X2S:A | Sclerotinia lectin like | β-trefoil | [PF14200](https://pfam.xfam.org/family/PF14200) | RicinB_lectin_2 |
| 2YIP:A | SML2 Micronemal protein | small protein / APPLE domain | [PF00024](https://pfam.xfam.org/family/PF00024) | PAN_1 |
| 2YIP:A | SML2 Micronemal protein | small protein / APPLE domain | [PF14295](https://pfam.xfam.org/family/PF14295) | PAN_4 |
| 1DQC:A | Invertebrate chitin-binding protein | small protein / Invertebrate chitin-binding protein | [PF01607](https://pfam.xfam.org/family/PF01607) | CBM_14 |
| 3A2E:A | Ginkbilobin | small protein / Knottin | [PF01657](https://pfam.xfam.org/family/PF01657) | Stress-antifung |
| 1MMC:A | hevein-like | small protein / Knottin | [PF00187](https://pfam.xfam.org/family/PF00187) | Chitin_bind_1 |
| 1QK7:A | Spider toxin | small protein / Knottin | [PF07740](https://pfam.xfam.org/family/PF07740) | Toxin_12 |

**Supplementary Table S4:** Species with the largest lectome and diversity in lectin classes

| **Super-kingdom** | **Kingdom** | **Phylum** | **Species** | **Description** | **number of lectin**  **classes for a score > 0.25** |
| --- | --- | --- | --- | --- | --- |
| Eukaryota | Metazoa | Brachiopoda | *Lingula unguis* | Primitive shell fish | 26 |
| Eukaryota | Metazoa | Chordata | *Branchiostoma floridae* | Fish: Florida lancelet | 24 |
| Eukaryota | Metazoa | Chordata | *Esox lucius* | Fish: Northern pike | 20 |
| Eukaryota | Metazoa | Cnidaria | *Acropora millepora* | Coral | 19 |
| Eukaryota | Metazoa | Cnidaria | A*ctinia tenebrosa* | Sea anemone | 23 |
| Eukaryota | Metazoa | Cnidaria | *Dendronephthya gigantea* | Coral | 24 |
| Eukaryota | Metazoa | Cnidaria | E*xaiptasia diaphana* | Sea anemone | 23 |
| Eukaryota | Metazoa | Cnidaria | *Orbicella faveolata* | Coral | 23 |
| Eukaryota | Metazoa | Cnidaria | *Pocillopora damicornis* | Coral | 20 |
| Eukaryota | Metazoa | Cnidaria | *Stylophora pistillata* | Coral | 22 |

**Supplementary Table S5:** Lectins from *Dendronephthya gigantea.*

|  | **# of prot** | **score** | **annotation** | **Binding site conservation** |
| --- | --- | --- | --- | --- |
| Ficolin-like | 166 | 0.66 | Ryncolin-1-like (fish toxin) | strong (NeuAc) |
| C-type lectin | 79 | 0.45 | E-selectin like | strong (Man, Fuc) |
| Pentraxin | 43 | 0.44 | neuronal pentraxin--like | low |
| F-type lectin | 33 | 0.48 | Fucolectin-like | strong (Fuc) |
| I-type lectin | 33 | 0.34 | cell adhesion molecule 2-like | low |
| L-rhamnose binding lectin | 31 | 0.49 | uncharacterized protein | medium (L-Rha) |
| H-type lectin | 13 | 0.45 | uncharacterized protein | strong (Gal) |
| Tectonin PropLec6B | 12 | 0.48 | uncharacterized protein | Strong (Methyl sugars) |
| Sea anemon lectin | 9 | 0.46 | uncharacterized protein | medium (lactose) |
| Trefoil Factor | 6 | 0.57 | integumentary mucin C.1-like | Strong |
| Cys-rich man-receptor | 6 | 0.43 | macrophage mannose receptor 1-like | medium |
| Ricin-like | 5 | 0.32 | polypeptide N-acetyl galactose aminyltransferase 13-like | low |
| Laminin G-like | 5 | 0.35 | laminin subunit alpha-4-like | low |
| P-type lectin | 4 | 0.42 | cation-independent mannose-6-phosphate receptor-like isoform X1 | low |
| Tachylectin-2-like PropLec5A | 4 | 0.35 | tachylectin-2-like | strong (GlcNAc) |
| Oyster_lectin | 4 | 0.28 | uncharacterized protein | strong (Man) |
| Mussel lectin | 3 | 0.37 | uncharacterized protein | medium |
| ERGIC-VIP L-type | 2 | 0.65 | protein ERGIC-53-like | Strong |
| P-type lectin-like | 2 | 0.51 | protein OS-9-like isoform X2 | Strong |
| P-domain of calnexin and reticulin | 2 | 0.73 | calreticulin-like | Strong |
| Malectin | 1 | 0.64 | malectin-like | Strong |
| Boletus and Laetiporus β-trefoil lectin | 1 | 0.27 | uncharacterized protein | Low |
| Yeast Emp L-type | 1 | 0.29 | protein ERGIC-53-like | Low |
| cyanovirin-like | 1 | 0.54 | uncharacterized protein | Medium |
